# High-Resolution Peripheral Quantitative Computed Tomography Can Assess Microstructural and Mechanical Properties of Human Distal Tibial Bone

**DOI:** 10.1359/jbmr.090822

**Published:** 2009-08-31

**Authors:** X Sherry Liu, X Henry Zhang, Kiranjit K Sekhon, Mark F Adams, Donald J McMahon, John P Bilezikian, Elizabeth Shane, X Edward Guo

**Affiliations:** 1 Bone Bioengineering Laboratory, Department of Biomedical Engineering, Columbia UniversityNew York, NY, USA; 2 Department of Applied Physics and Applied Mathematics, Columbia UniversityNew York, NY, USA; 3 Division of Endocrinology, Department of Medicine, Columbia UniversityNew York, NY, USA

**Keywords:** bone microstructure, bone stiffness, finite element model, high-resolution peripheral quantitative computed tomography, micro-computed tomography

## Abstract

High-resolution peripheral quantitative computed tomography (HR-pQCT) is a newly developed in vivo clinical imaging modality. It can assess the 3D microstructure of cortical and trabecular bone at the distal radius and tibia and is suitable as an input for microstructural finite element (µFE) analysis to evaluate bone's mechanical competence. In order for microstructural and image-based µFE analyses to become standard clinical tools, validation with a current gold standard, namely, high-resolution micro-computed tomography (µCT), is required. Microstructural measurements of 19 human cadaveric distal tibiae were performed for the registered HR-pQCT and µCT images, respectively. Next, whole bone stiffness, trabecular bone stiffness, and elastic moduli of cubic subvolumes of trabecular bone in both HR-pQCT and µCT images were determined by µFE analysis. The standard HR-pQCT patient protocol measurements, derived bone volume fraction (BV/TV^d^), trabecular number (Tb.N*), trabecular thickness (Tb.Th), trabecular spacing (Tb.Sp), and cortical thickness (Ct.Th), as well as the voxel-based direct measurements, BV/TV, Tb.N*, Tb.Th*, Tb.Sp*, Ct.Th, bone surface-to-volume ratio (BS/BV), structure model index (SMI), and connectivity density (Conn.D), correlated well with their respective gold standards, and both contributed to µFE-predicted mechanical properties in either single or multiple linear regressions. The mechanical measurements, although overestimated by HR-pQCT, correlated highly with their gold standards. Moreover, elastic moduli of cubic subvolumes of trabecular bone predicted whole bone or trabecular bone stiffness in distal tibia. We conclude that microstructural measurements and mechanical parameters of distal tibia can be efficiently derived from HR-pQCT images and provide additional information regarding bone fragility. © 2010 American Society for Bone and Mineral Research.

## Introduction

Osteoporosis, a prevalent metabolic bone disease, is characterized by low bone mass and microarchitectural deterioration.(1) Measurement of areal bone mineral density (aBMD) by dual-energy X-ray absorptiometry (DXA) is currently the only validated method for diagnosis of osteoporosis and assessment of fracture risk in postmenopausal women and men over age 50.(2) It is becoming increasingly apparent, however, that other factors, independent of aBMD, contribute significantly to fracture risk.(3) Along with clinical risk factors such as age and a previous fracture, skeletal properties of trabecular microstructure, cortical thickness and porosity, and bone geometry are key independent determinants. Together these skeletal features contribute to bone's biomechanical properties such as elastic stiffness and failure load.(4–10)

Recently, an in vivo clinical imaging modality, high-resolution peripheral quantitative computed tomography (HR-pQCT), has been developed to assess bone microstructure (XtremeCT, Scanco Medical AG, Bassersdorf, Switzerland). The 3D data sets provided by HR-pQCT permit separate analyses of trabecular and cortical bone at peripheral sites (distal tibia and distal radius).(11) Although HR-pQCT was developed recently, its ability to detect age- or disease-related changes in bone microarchitecture and to provide additional fracture risk determinants has been demonstrated in several clinical studies.(3,11–15) Boutroy and colleagues first reported that HR-pQCT can discriminate between osteopenic postmenopausal women with or without fractures and that those with fractures have decreased trabecular BMD and increased variability in trabecular spacing.(11) Sornay-Rendu and collagues reported that architectural alterations of trabecular and cortical bone of postmenopausal women assessed by HR-pQCT are associated with vertebral and nonvertebral fractures and are partially independent of decreased BMD.(3) These studies confirmed the importance of microstructural measurements as additional indicators of bone fragility and suggested that HR-pQCT is likely to inaugurate a new era of noninvasive quantitative skeletal imaging.

HR-pQCT images also can be used for building microstructural finite element (µFE) models to assess bone strength, a measurement of bone's resistance to fractures. µFE analysis can be performed either on whole bone segments to obtain the axial stiffness or strength(10,13,16,17) or on trabecular bone subvolumes to determine the elastic moduli.(10,18,19) Melton and colleagues showed that in addition to BMD, derived bone strength parameters (e.g., axial rigidity and fall load to failure load ratio), bone geometry, and microstructure are determinants of forearm fracture risk prediction.(17) Boutroy and colleagues found that the proportion of the load carried by trabecular bone versus cortical bone is associated with wrist fracture independent of BMD and microarchitecture.(13) These clinical studies demonstrate that HR-pQCT-based µFE analyses can provide measurements of mechanical properties that associate independently with fracture risk.

The resolution of HR-pQCT, while allowing visualization of the internal structure of trabecular bone, is very close to the low end of trabecular thickness, and thus insufficient resolution may influence microstructural measurements and image-based µFE predictions. In order for microstructural and image-based µFE analyses to become standard clinical tools, the data from HR-pQCT must be thoroughly validated and compared with a current gold standard, namely, high-resolution micro-computed tomography (µCT). Only a few ex vivo validation studies have compared HR-pQCT and µCT images of the distal radius.(15,16,18,20) However, because of the large size of the distal tibia, there are no data validating HR-pQCT of the distal tibia against the gold standard µCT. Nevertheless, there are significant differences between the geometry and microstructure of the distal tibia and the distal radius.(11,18,21) Thus conclusions established in a validation study of the distal radius might not be valid for the distal tibia, and a validation study for distal tibia against the gold standard µCT is necessary.

In this study, the primary purpose was to validate the standard HR-pQCT microstructural measurements from the patient evaluation protocol against gold standard µCT measurements of the distal tibia. The second purpose was to compare the microstructural measurements of HR-pQCT using a direct method normally used for µCT images against gold standard µCT measurements. To fulfill the second objective, images that output from a standard HR-pQCT filtering and thresholding protocol were used. Subsequently, the influences of different morphologic analysis techniques on accuracy of microstructural measurements were studied. Further, the axial stiffness of the whole distal tibial bone segment, with and without cortex, as assessed by HR-pQCT-based µFE models, was validated against the gold standard, µCT-based µFE measurements. Then the elastic moduli derived by HR-pQCT-based µFE analysis of a cubic subvolume of trabecular bone were validated by their respective gold standards and then correlated with the whole bone stiffness by µCT-based µFE analysis to assess whether the elastic moduli of the cubic subvolume reflects the mechanical competence of the whole bone segment. Lastly, the ability of HR-pQCT microstructural measurements to predict the mechanical properties calculated by µCT-based µFE analysis was investigated. This represents the first validation study for HR-pQCT-based microstructural and mechanical measurements of human distal tibia.

## Materials and Methods

### Specimen preparation and HR-pQCT and µCT scans

Nineteen freshly frozen human cadaveric tibiae from 13 donors (6 pairs and 7 singles, 10 males and 3 females) were obtained from the International Institute for the Advancement of Medicine (Scranton, PA, USA). The age of subjects ranged from 55 to 84 years, with an average of 70.6 years. The subjects' medical histories were screened to exclude metabolic bone diseases or bone cancer. The whole tibiae were scanned first by HR-pQCT (XtremeCT, Scanco Medical AG, Bassersdorf, Switzerland) with the same settings used clinically (60 kVp, 1000 µA, 100 ms integration time). A reference line was manually placed at the endplate of the tibia to select the region of interest in the anteroposterior scout view. The HR-pQCT measurement included 110 slices, corresponding to a 9.02 mm section along the axial direction. Using a band saw, each distal tibia was cut into a 25 mm section along the axial direction centered by the scanned area. The central 10 mm section along the axial direction then was scanned by µCT (µCT 80, Scanco Medical AG) to encompass the same region scanned by HR-pQCT. An ex vivo scanning setting (70 kVp, 114 µA, 700 ms integration time) was used for µCT scanning, resulting in an isotropic 25 µm voxel size.

### Mutual information-based registration of HR-pQCT and µCT images

**Fig. 1:**
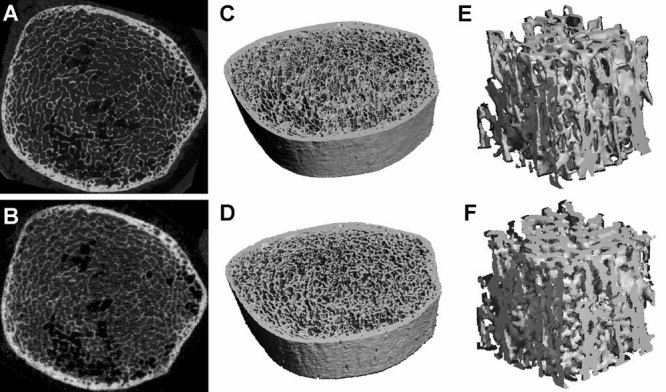
The registered 2D gray-scale image (*A*, *B*), 3D thresholded image (*C*, *D*), and 3D cubic subvolume (*E*, *F*) of HR-pQCT and µCT images of human distal tibia. The first row shows the high-resolution µCT images, and the second row shows the corresponding HR-pQCT images.

To register µCT images to HR-pQCT images, a pyramidal three-step registration approach was employed using a landmark-initialized mutual information-based registration toolkit(22,23) of an open-source software (National Library of Medicine Insight Segmentation and Registration Toolkit, Clifton Park, NY, USA).(24) The gray-scale HR-pQCT images were fixed, whereas the gray-scale µCT images were transformed to match the fixed images. To test the registration accuracy, three µCT images of 25 µm voxel size were first transformed with user-specified translation (1 mm), rotation (45 degrees), or combination of both and then resampled to 82 µm voxel size, the typical voxel size of the HR-pQCT image. The original µCT images were registered to the simulated HR-pQCT images, and the registration results were compared with the transformation parameters. Results showed that the tested image sets were registered with an alignment error smaller than 10% of the µCT voxel size. All the µCT images were registered successfully to the corresponding HR-pQCT images to encompass the same volume of interest (see Fig. 1) and confirmed by visual inspection using an open-source medical image display application FusionViewer.(25)

### Morphologic analyses for µCT images

The standard µCT evaluation protocol was performed for the registered µCT images. First, the endosteal cortical surface was drawn manually for every fifth slice. Then, by using a “morph” function of the Scanco software, the contours of the neighboring four slices were created automatically. Subsequently, each software-generated contour was checked carefully, and small changes were made when necessary to ensure the accuracy of each contour. After manual segmentations of the trabecular and cortical region, the trabecular bone region was processed by Gaussian filtering and specimen-specific adaptive thresholding to extract the mineralized phase using the standard protocol of Scanco software for µCT analysis. Based on the thresholded trabecular bone image, bone volume fraction (BV/TV) was determined by dividing the total bone voxel volume (BV) by the total volume (TV) of interest. Trabecular thickness (Tb.Th*) was evaluated via a distance transformation of the 3D bone image and then determined by the average diameter of the maximum spheres that fit inside the bone tissue.(26) Trabecular spacing (Tb.Sp*) was computed in a similar fashion, where the distance transformation was instead applied to the bone marrow space. Trabecular number (Tb.N*) was determined as the inverse of the mean distance between the midline of trabecular bone tissue. In addition, bone surface-to-volume ratio (BS/BV), structure model index (SMI), connectivity density (Conn.D), and degree of anisotropy (DA) also were evaluated for each trabecular bone image. BS/BV was computed by dividing the trabecular bone surface (BS) by the trabecular bone volume (BV), with BS and BV determined by a triangulation approach.(27) SMI estimates, on average, the plate versus rod characteristics of trabecular bone.(21,28–30) It is defined as SMI = 6(BV/BS^2^)[*d*(BS)/*dr*], where *d*(BS)/*dr* is the surface area derivative with respect to *r*, the half-thickness or radius of the microstructure. SMI ranges from 0 for an ideal plate structure to 3 for an ideal rod structure. Conn.D quantifies trabecular connectivity by calculating the number of handles or closed loops in a trabecular network.(31,32) DA reflects the orientation of the trabecular bone network. To determine the DA, first, a fabric tensor (3 × 3 matrix) with the eigenvectors representing the main directions of structure, and the eigenvalues indicating the inclination of structure toward the main directions were calculated.(33,34) Then DA was calculated as the ratio of the maximal and minimal eigenvalues. To evaluate cortical thickness (Ct.Th), both periosteal and endosteal surfaces of the tibial cortex were contoured manually using the semiautomatic Scanco software to isolate the cortex as the volume of interest. The Ct.Th was determined as the cortical volume divided by the mean of periosteal and endosteal bone surface area according to a standard Scanco protocol for µCT images. All voxel-based direct morphologic analyses for µCT images were performed using the standard morphologic analysis software on an HP AlphaStation of a VivaCT 40 system (Scanco Medical AG).

### Morphologic analyses for HR-pQCT images

All HR-pQCT images were analyzed by two evaluation protocols. The standard patient evaluation for HR-pQCT images (indicated as patient analysis) was applied first, followed by the standard analysis protocol for µCT images (indicated as direct analysis), as described in the preceding section.

According to the standard patient evaluation protocol, the periosteal surface of the tibia was contoured first semiautomatically and then by an automated threshold-based algorithm to separate the cortical and trabecular compartments.(35) Therefore, the entire trabecular and cortical regions with irregular shape and size were segmented for each tibia. Attenuation values were converted to hydroxyapatite density (mg HA/cm^3^) using a linear conversion. The mineralized phase was thresholded automatically by using a Laplace-Hamming filter followed by a global threshold using a fixed value of 40% of maximal gray-scale value of the images.(18) Because the spatial resolution of HR-pQCT may not be high enough to depict the true individual thicknesses of the trabeculae, a semi-derived morphologic analysis instead of a voxel-based analysis was developed for assessing the microstructure of trabecular bone based on gray-scale HR-pQCT images.(35,36) Trabecular bone density (*D*_trab_) in mg HA/cm^3^ was calculated as the average mineral density within the trabecular region. The relative bone volume fraction (BV/TV^d^) was calculated from *D*_trab_, assuming a density of fully mineralized bone of 1200 mg/cm^3^ [i.e., BV/TV^d^ = *D*_trab_ (mg HA/cm^3^)/1200 mg HA/cm^3^].(11,35) To assess trabecular microstructure, 3D ridges (the center points of trabeculae) were identified, and the spacing between them was assessed three-dimensionally by the distance transformation method.(26,35–37) Trabecular number (Tb.N*) was defined as the inverse of the mean spacing of the 3D ridges. This procedure is truly 3D and model-independent. Trabecular thickness (Tb.Th) and spacing (Tb.Sp) then were derived from BV/TV^d^ and directly measured Tb.N* [i.e., Tb.Th^d^ = BV/TV^d^/Tb.N* and Tb.Sp^d^ = (1 – BV/TV^d^)/Tb.N*] by analogy to standard histomorphometry.(38) For the cortical region, mean cortical thickness (Ct.Th) was assessed as the cortical volume divided by the outer bone surface according to the standard patient evaluation protocol of the manufacturer. All the morphologic analyses from the HR-pQCT patient evaluation protocol were performed using Scanco software installed on an HP AlphaStation operating in a VMS environment (Hewlett-Packard, Palo Alto, CA, USA).

Next, voxel-based direct morphologic analysis was applied to the thresholded trabecular bone image output from the HR-pQCT patient evaluation. BV/TV, Tb.N*, Tb.Th*, Tb.Sp*, Ct.Th, SMI, BS/BV, DA, and Conn.D of each HR-pQCT image were calculated. The methods of these analyses are the same as the standard morphologic analysis for µCT images, as described in the preceding section.

### Finite element analyses for HR-pQCT and µCT images

Three µFE analyses were performed for each HR-pQCT and µCT image. The first analysis was applied to the whole tibial bone segment to determine its axial stiffness. The second analysis was applied to the tibial trabecular bone segment without cortex to determine the trabecular bone axial stiffness. The third analysis was applied to a cubic subvolume of trabecular bone selected from the center of the sample to calculate the anisotropic elastic moduli. These three µFE analyses provide distinct mechanical parameters of the distal tibia that are important in clinical assessments of mechanical competence. The axial stiffness is the overall mechanical competence, whereas trabecular bone axial stiffness provides an estimation of the overall structural contributions of the trabecular bone compartment. The subvolume trabecular bone µFE analysis reveals important anisotropic material properties of the trabecular bone component. Each bone voxel of a thresholded HR-pQCT image was converted directly to an eight-node elastic brick element with element size 82 × 82 × 82 µm^3^. Each µCT image was resampled from 25 to 40 µm and then converted to µFE models with element size 40 × 40 × 40 µm^3^. A convergence study was conducted in three randomly selected µCT images to determine the axial stiffness with different element size (25, 30, 40, 60, and 80 µm). Results showed that the maximum difference in axial stiffness between the models constructed at 40 and 25 µm was 1.67%. For each µFE analysis, bone tissue was modeled as an isotropic linear elastic material with a Young's modulus (*E*_*s*_) of 15 GPa and a Poisson's ratio of 0.3.(39)

To evaluate the axial stiffness for a whole bone segment with and without the cortical shell, a uniaxial displacement equaling 1% of the bone-segment height was applied perpendicularly to the distal surface of the tibia while the proximal surface was imposed with zero displacement along the same direction. Both ends of the tibia were allowed to expand freely in the transverse plane. The total reaction force was calculated from the linear µFE analysis, and the axial stiffness was calculated as the reaction force divided by the imposed displacement. Typically, there were about 5 million elements and 8 million nodes in the µFE model derived from an HR-pQCT image and 40 million elements and 60 million nodes in the model from a µCT image. The parallel µFE program Olympus, which is built on a serial finite element analysis program FEAP,(37) a parallel multigrid equation solver Prometheus,(40) and the parallel numerical framework PETSc,(41) was used to solve these µFE models.(42) All the parallel computations were conducted on an IBM Power4 supercomputer (IBM Corporation, Armonk, NY, USA) at the San Diego Supercomputer Center using a maximum of 256 CPUs in parallel. Whole bone segment stiffness and trabecular bone stiffness were calculated for each HR-pQCT and µCT image, respectively.

A 143 × 143 × 143 voxel cubic subvolume of trabecular bone was extracted from each registered and resampled µCT image (40 µm voxel size) corresponding to a 70 × 70 × 70 voxel cubic subvolume (82 µm voxel size) from the HR-pQCT image (see Fig. 1) equivalent to a physical size of 5.74 × 5.74 × 5.74 mm^3^. The subvolumes from two modalities were converted automatically to µFE models. Using a customized element-by-element preconditioned conjugate gradient solver,(43) six µFE analyses were performed for each model, representing three uniaxial compression tests along three imaging axes (*x*, *y*, and *z*) and three uniaxial shear tests.(44) The general anisotropic stiffness matrix was first determined based on the results from the preceding analyses. A new coordinate system of orthotropic axes (*X*_1_, *X*_2_, and *X*_3_) representing the best orthotropic symmetry then was calculated by using a numerical optimization algorithm, Powell's method,(45) to minimize an orthotropy objective function.(44) The transformation of the anisotropic matrix to a new coordinate system yielded the full orthotropic stiffness tensor.(44) The elastic constants and stiffness matrix were sorted such that *E*_11_ was in the direction of the lowest axial modulus and *E*_33_ was in the direction of the highest axial modulus. The elastic moduli (three Young's moduli, *E*_11_ < *E*_22_ < *E*_33_, and three shear moduli, *G*_23_, *G*_31_, *G*_12_) then were derived from the orthotropic stiffness tensor. All the µFE analyses for subvolumes were implemented on a Dell XPS PC workstation (Dell, Inc., Round Rock, TX, USA).

### Statistical analysis

Linear correlations between the HR-pQCT microstructural measurements and the corresponding measurements from gold standard µCT images were performed. The correlation coefficients and slopes of two linear correlations, HR-pQCT patient analysis measurements versus µCT gold standard measurements and HR-pQCT direct analysis measurements versus µCT gold standard measurements, were compared statistically.(46) Linear correlations also were conducted to compare the mechanical measurements of HR-pQCT and µCT images, including whole bone segment stiffness, trabecular bone stiffness, and six elastic moduli of subvolumes. Paired Student's *t* tests were performed to test for significant differences between corresponding HR-pQCT and µCT measurements. In addition, all the mechanical measurements were compared between two modalities through an analysis of covariance (ANCOVA) with repeated measures and the BV/TV as the covariate.

Furthermore, each of the microstructural measurements of HR-pQCT and µCT was correlated individually with the mechanical properties derived from the µCT-based µFE model by linear regression. Next, forward stepwise multiple linear regression was performed to predict mechanical properties by the microstructural measurements of HR-pQCT (BV/TV^d^, Tb.N*, Tb.Th, Tb.Sp, and Ct.Th from patient analysis and BS/BV, SMI, Conn.D, and DA from direct analysis) and µCT (BV/TV, Tb.N*, Tb.Th*, Tb.Sp*, Ct.Th, BS/BV, SMI, Conn.D, and DA), respectively. At each step of the forward stepwise regression method, the eligible independent variable with the highest statistical strength entered the model. At any subsequent step where two or more independent variables were selected into the model, the variable with the least statistical strength was removed from the model. The stepping was terminated when no eligible independent variable exceeded the critical value (*p* < .05) for model entry or when no independent variable in the model reached the standard (*p* > .1) for variable removal. The significant and independent predictors of microstructure parameters were selected to yield the best prediction of each calculated mechanical parameter. In addition, each elastic modulus derived from HR-pQCT and µCT subvolumes was correlated with the whole bone stiffness and trabecular bone stiffness derived from µCT images by linear regression.

The stepwise multiple linear regressions were performed by SPSS 13.0 software (SPSS, Inc., Chicago, IL, USA). All other statistical analyses were performed using KaleidaGraph 3.6 software (Synergy Software, Reading, PA, USA).

## Results

On qualitative inspection of the HR-pQCT and the registered µCT images, similar trabecular bone patterns were found; however, high-resolution µCT images showed finer trabeculae and more detailed local trabecular bone morphology than HR-pQCT images (Fig. 1).

**Fig. 2:**
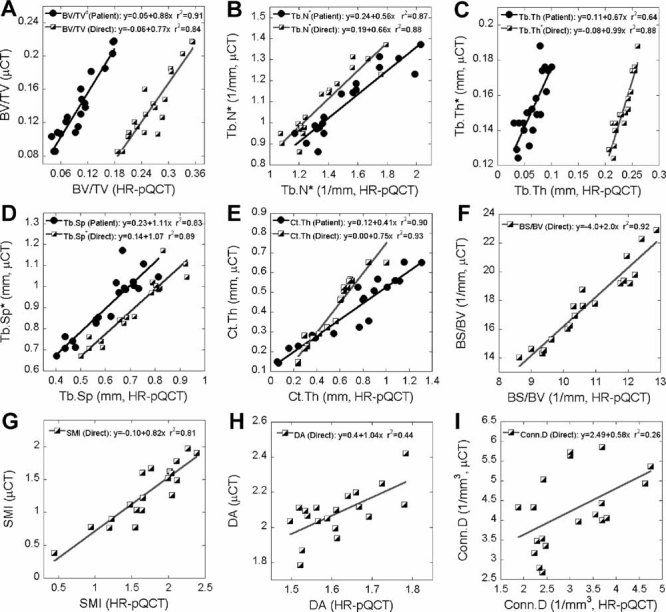
Correlations between microstructural measurements of HR-pQCT by patient and direct analysis and their respective µCT gold standards.

**Fig. 3:**
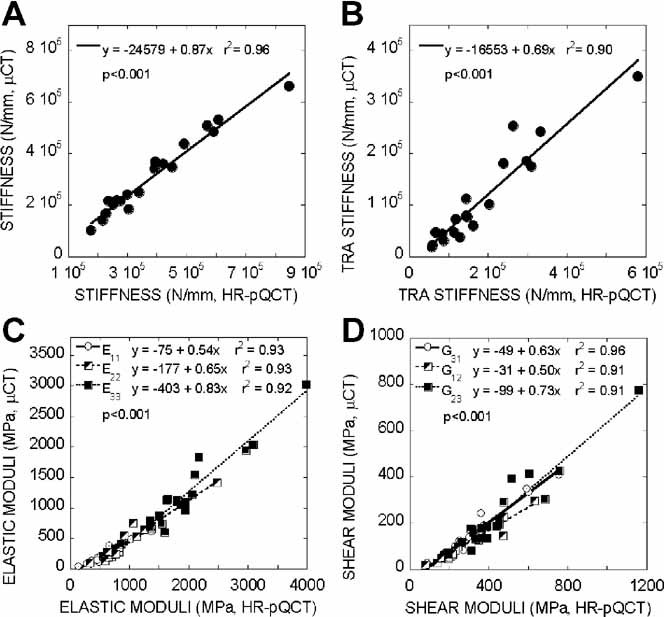
Correlation between (*A*) whole bone stiffness, (*B*) trabecular bone stiffness, (*C*) elastic moduli, and (*D*) shear moduli of registered subvolumes of HR-pQCT and gold standard µCT images.

The mean and standard deviation of microstructural and mechanical measurements of HR-pQCT and µCT images are presented in Table 1 and 2. All the HR-pQCT microstructural measurements were significantly different from the corresponding µCT measurements (*p* < .05; see Table 1). Both the HR-pQCT patient protocol measurements (BV/TV^d^, Tb.N*, Tb.Th, Tb.Sp, and Ct.Th; *r*^2^ = 0.64–0.91) and direct measurements (BV/TV, Tb.N*, Tb.Th*, Tb.Sp*, and Ct.Th; *r*^2^ = 0.84–0.93) correlated significantly and highly with their respective gold standard measurements (BV/TV, Tb.N*, Tb.Th*, Tb.Sp*, and Ct.Th; Fig. 2). For each measurement, the correlation coefficient (*r*) between patient HR-pQCT and gold standard measurement and between direct HR-pQCT and gold standard measurement were not statistically different (*p* > .05). However, the slopes of each pair of correlations were statistically different (*p* < .05). Significant correlations also were found for BS/BV (*r*^2^ = 0.92), SMI (*r*^2^ = 0.81), DA (*r*^2^ = 0.46), and Conn.D (*r*^2^ = 0.26) of HR-pQCT and µCT images. In general, HR-pQCT-based µFE analyses overestimated all the mechanical measurements (*p* < .05; Table 2) but significantly and strongly correlated with their gold standards (*r*^2^ = 0.91–0.96; Fig. 3) The ANCOVA test with repeated measures showed that all the mechanical measurements were significantly different between the two modalities even with removal of variance attributable to BV/TV. The orthotropic axes in the principle coordinates system of trabecular bone subvolumes derived from HR-pQCT-based µFE analysis also agreed well with those from µCT. The angle between the longitudinal axes of µCT and HR-pQCT images was 1 ± 0.5 degrees, and the angle between the transverse axes of µCT and HR-pQCT images was 5.5 ± 8.5 degrees.

**Table 1:** Microstructural Measurements of HR-pQCT and µCT Images of Distal Tibiae

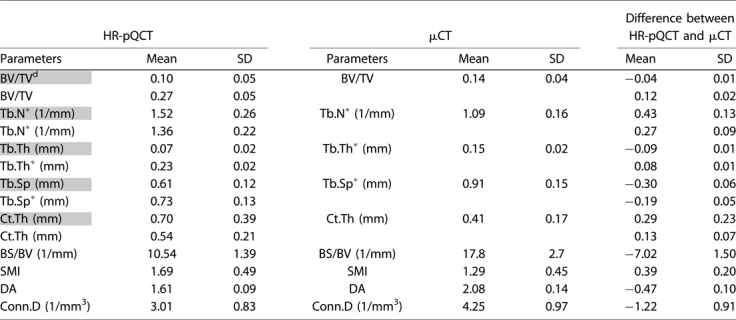

Parameters highlighted in gray represent the HR-pQCT patient protocol measurements. Others represent the direct measurements from µCT standard evaluation protocol. SD = standard deviation. All the measurements of HR-pQCT are significantly different from those of µCT indicated by paired Student's *t* tests (*p* < .05).

**Table 2:** Mechanical Measurements of HR-pQCT and µCT Images of Distal Tibiae

	HR-pQCT		µCT		Difference between HR-pQCT and µCT	
			
Parameter	Mean	SD	Mean	SD	Mean	SD
Whole bone stiffness (N/mm)	388,517	172,277	313,691	152,993	74,826	37,479
Bone volume of whole bone µFE model (mm^3^)	2,011	686	1,435	540	576	178
Trabecular bone stiffness (N/mm)	187,063	128,778	111,868	93,303	75,195	50,194
Bone volume of trabecular bone µFE model (mm^3^)	1258	511	701	340	557	197
*E*_11_ (MPa)	541	296	218	166	323	143
*E*_22_ (MPa)	892	460	400	309	492	181
*E*_33_ (MPa)	1960	716	1224	621	736	215
*G*_23_ (MPa)	463	219	240	168	223	76
*G*_31_ (MPa)	314	167	150	108	164	65
*G*_12_ (MPa)	260	138	98	72	162	72
BV/TV of cubic subvolume µFE model	0.25	0.06	0.14	0.05	0.11	0.01

SD = standard deviation. All the measurements of HR-pQCT are significantly different from those of µCT indicated by paired Student's *t* tests (*p* < .05).

The trabecular microstructure measurements from the HR-pQCT patient analysis (BV/TV^d^, Tb.N*, Tb.Th, and Tb.Sp), HR-pQCT direct analysis (BV/TV, Tb.N*, Tb.Th*, and Tb.Sp*), and their µCT gold standards (BV/TV, Tb.N*, Tb.Th*, and Tb.Sp*) were highly correlated with whole bone stiffness, trabecular bone stiffness, and elastic moduli of distal tibiae (*p* < .01; Table 3). Results for Ct.Th from both HR-pQCT and µCT did not contribute to stiffness or moduli measurements. BS/BV (*r*^2^ = 0.43–0.59), SMI (*r*^2^ = 0.34–0.72), and Conn.D (*r*^2^ = 0.44–0.64) of HR-pQCT correlated significantly with stiffness and elastic moduli measurements (*p* < .01), whereas DA of HR-pQCT showed no contribution (*p* > .05). Of the gold standard µCT measurements, BS/BV (*r*^2^ = 0.51–0.65) and SMI (*r*^2^ = 0.49–0.81) correlated significantly with stiffness and moduli (*p* < .001); however, no correlation was found for DA and Conn.D (*p* > .05).

**Table 3:** Correlation (*r*^2^) Between the Microstructural Measurements of HR-pQCT and µCT Images and the Mechanical Measurements of µCT Images

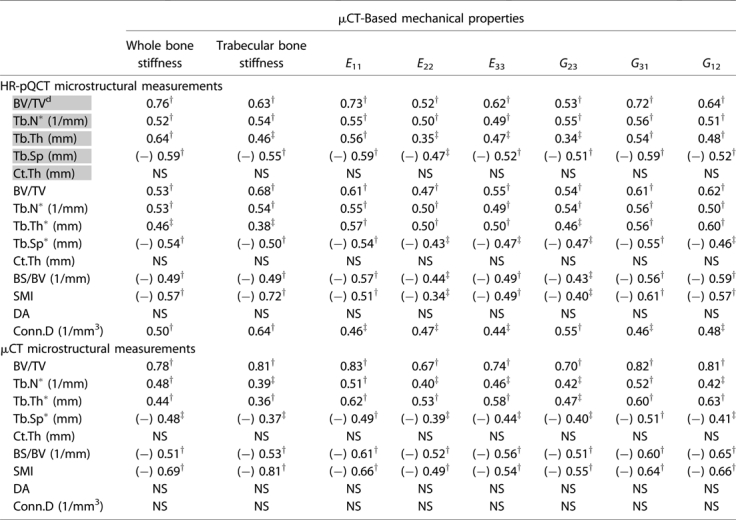

Parameters highlighted in gray represent the HR-pQCT patient protocol measurements. Others represent the direct measurements from µCT standard evaluation protocol. (−) indicates negative correlation.

† *p* < .001

‡ *p* < .01; NS: *p* > .05.

The results from the multiple linear regression analyses suggested that BV/TV^d^ by HR-pQCT and BV/TV by µCT were the most important predictors of the mechanical measurements (Table 4). For prediction of whole bone stiffness and *E*_11_, *E*_22_, *E*_33_, *G*_31_, and *G*_12_ of bone subvolumes, BV/TV^d^ by HR-pQCT and BV/TV by µCT were the only independent and significant predictors. For trabecular bone stiffness, SMI with negative coefficient and Conn.D by HR-pQCT were the two independent predictors; however, from µCT measurements, SMI appeared to be the only significant predictor. Furthermore, Tb.N* and BS/BV by HR-pQCT significantly predicted *G*_23_ independent of other parameters, whereas BV/TV was the only significant predictor for *G*_23_ among µCT microstructural measurements (see Table 4).

**Table 4:** Correlation (Adjusted r^2^) and Independent Predictors of Multilinear Regression for Prediction of the Gold Standard Mechanical Measurements by the Microstructural Measurements of HR-pQCT and µCT

	HR-pQCT				µCT			
		
µCT-based mechanical properties	Independent predictors	Constant	Coefficient	Adjusted *r*^2^	Independent predictors	Constant	Coefficient	Adjusted *r*^2^
Whole bone stiffness	BV/TV^d^	13,206	2,927,806	0.74	BV/TV	−136,253	3,189,905	0.76
Trabecular stiffness	SMI	147,495	−110,063	0.84	SMI	354,107	−188,494	0.80
	Conn.D		49,892					
*E*_11_	BV/TV^d^	−102	3,119	0.71	BV/TV	−289	3,591	0.82
*E*_22_	BV/TV^d^	−103	4,902	0.49	BV/TV	−442	5,975	0.65
*E*_33_	BV/TV^d^	120	10,759	0.60	BV/TV	−558	12,639	0.72
*G*_23_	Tb.N*	248	361	0.62	BV/TV	−119	1,538	0.80
	BS/BV		−52					
*G*_31_	BV/TV^d^	−57	2,019	0.70	BV/TV	−232	3,344	0.69
*G*_12_	BV/TV^d^	−31	1,265	0.62	BV/TV	−179	2,333	0.82

The order of predictors is the order in which predictors enter the stepwise regression model. All the predictions are statistically significant with *p* < .001.

Elastic moduli derived from the HR-pQCT subvolume correlated significantly with whole bone stiffness (*r*^2^ = 0.42–0.54, *p* < .01) and trabecular bone stiffness (*r*^2^ = 0.50–0.65, *p* < .001; see Table 5). Similar predictive power was found for moduli of µCT subvolume to whole bone stiffness (*r*^2^ = 0.48–0.60, *p* < .001) and trabecular bone stiffness (*r*^2^ = 0.53–0.70, *p* < .001; see Table 5).

**Table 5:** Correlation (*r*^2^) Between the Elastic Moduli of HR-pQCT and µCT Subvolumes and the Whole Bone Stiffness and Trabecular Bone Stiffness of µCT Images

	HR-pQCT-based parameters						µCT-based parameters					
		
µCT-based parameters	*E*_11_	*E*_22_	*E*_33_	*G*_23_	*G*_31_	*G*_12_	*E*_11_	*E*_22_	*E*_33_	*G*_23_	*G*_31_	*G*_12_
Whole bone stiffness	0.53†	0.43‡	0.42‡	0.48†	0.54†	0.53†	0.59†	0.48†	0.50†	0.50†	0.58†	0.60†
Trabecular bone stiffness	0.61†	0.56†	0.50†	0.65†	0.61†	0.61†	0.61†	0.53†	0.53†	0.62†	0.61†	0.70†

† *p* < .001;

‡ *p* < .01.

## Discussion

In this study, ex vivo microstructural and mechanical measurements of the newly developed HR-pQCT scan of the distal tibia were validated against the gold standard, high-resolution µCT. Moreover, we compared the HR-pQCT microstructural measurements from the patient protocol in clinical use and from those adopted from the µCT evaluation protocol and evaluated the contributions of these microstructural measurements to the mechanical competence of the distal tibia. We found that the microstructural measurements from the clinical HR-pQCT protocol correlated significantly with their respective gold standards (*r*^2^ = 0.64–0.91), with the highest correlation in BV/TV^d^ and lowest in Tb.Th. These relationships are consistent with previous observations on the distal radius (*r*^2^ = 0.59–0.96).(18) However, the absolute values of the HR-pQCT measurements differ significantly from the gold standard (see Table 1). Because HR-pQCT underestimates BV/TV^d^ and overestimates Tb.N*, both derived parameters, Tb.Th (Tb.Th = BV/TV^d^/Tb.N*) and Tb.Sp [Tb.Sp = (1 – BV/TV^d^)/Tb.N*] were significantly underestimated. In addition, HR-pQCT overestimates Ct.Th likely because of partial-volume effects. Compared with the semiderived patient protocol measurements (BV/TV^d^, Tb.N*, Tb.Th, Tb.Sp, and Ct.Th; *r*^2^ = 0.64–0.91), direct measurements of BV/TV, Tb.N*, Tb.Th*, Tb.Sp*, and Ct.Th of HR-pQCT images (*r*^2^ = 0.84–0.93) did not have improved correlations with the gold standards. Therefore, to increase the accuracy of the HR-pQCT measurements, an improvement of image-preprocessing (image acquiring, filtering, and thresholding) techniques might be more important than that of microstructural measurement techniques. Among all the direct microstructural measurements, high correlations between HR-pQCT and µCT were found for most parameters (BV/TV, Tb.N*, Tb.Th*, Tb.Sp*, Ct.Th, BS/BV, and SMI; *r*^2^ = 0.81–0.93) except for DA (*r*^2^ = 0.46) and Conn.D (*r*^2^ = 0.26). These results differ from those reported for the distal radius, which showed high correlations for most parameters, including Conn.D (*r*^2^ = 0.91) and DA (*r*^2^ = 0.61) but low or no correlations for Tb.Th*, BS/BV, and SMI.(18) The discrepancies between our results and those reported previously for the distal radius are likely due to the dramatic difference in trabecular morphology between the two sites: The distal tibia in the current study tended to have a more platelike structure and less separated trabeculae than the distal radius, as noted in the study by MacNeil and Boyd.(18) In addition, the input image for these voxel-based direct measurements resulted from a global threshold technique following the patient evaluation protocol. A previous study demonstrated that global threshold could have negative influences on voxel-based measurements and suggested that by adopting a more advanced local threshold method, the accuracy of these measurements could be improved.(47)

Although the mechanical measurements by HR-pQCT-based µFE analyses significantly overestimated those by µCT-based µFE analyses (see Table 2), high correlations were found when both were compared (see Fig. 3). Correlations between HR-pQCT- and µCT-based whole bone stiffness, trabecular bone stiffness, and six elastic moduli of trabecular subvolumes were highly significant, with *r*^2^ > 0.90. By visual comparison of the registered HR-pQCT and µCT images, the thresholded HR-pQCT image contained thicker trabeculae but otherwise resembled the gold standard µCT images (see Fig. 1). This could explain the overestimate of the mechanical measurements despite the significantly high correlation of HR-pQCT with the gold standards. However, even with removal of variance attributable to difference in BV/TV, the mechanical measurements based on two modalities were still significantly different. Once again, the current threshold technique from the HR-pQCT patient evaluation protocol affected the accuracy of voxel-based analyses, including the µFE analysis. By adopting a more advanced threshold technique,(47) the overestimation of mechanical measurements could be corrected.

The ability of HR-pQCT-based microstructural measurements to indicate the mechanical measurements of the distal tibia is comparable with that of µCT (see Table 3). The measurement with the highest prediction power was BV/TV^d^; in contrast, Ct.Th and DA of HR-pQCT did not correlate with mechanical competence of the distal tibia. In multiple linear regressions, BV/TV^d^ was the only independent predictor for most mechanical measurements. For prediction of trabecular bone stiffness, a combination of SMI and Conn.D resulted in the highest prediction power, suggesting that trabecular bone stiffness increases with more plate-like trabeculae and more intact structure, as would be expected. It is interesting that Ito and colleagues identified that SMI is the strongest predictor for vertebral fractures among all the measurements, including BMD and BV/TV.(48) Tb.N* and BS/BV were the only independent predictors of *G*_23_ by HR-pQCT. Tb.Th and Tb.Sp, as measured by HR-pQCT, which depend on the measurements of BV/TV^d^ and Tb.N*, did not independently increase the predictive power of any mechanical measurement. Similarly, neither Tb.Th* nor Tb.Sp*, which are direct and model-independent µCT measurements, showed any additional contribution to the prediction of mechanical properties of the distal tibia. MacNeil and Boyd, who tested the ability of HR-pQCT measurements to predict mechanical properties derived by HR-pQCT-based µFE analysis of the distal tibia and distal radius, found that trabecular and cortical BMD, cross-sectional area, BV/TV, and Ct.Th are the most critical predictors.(10) Our results suggest that among the microstructural measurements of HR-pQCT, BV/TV^d^ is the most important indicator for mechanical competence of the distal tibia. In addition to BV/TV^d^, Tb.N*, BS/BV, SMI, and Conn.D independently contribute to the mechanical properties of trabecular bone.

It is important to evaluate whole bone stiffness of the distal tibia based on HR-pQCT and µFE analysis because it correlates significantly with ultimate load, a parameter that indicates fracture resistance.(16) However, the large model size for whole bone analysis limits the ease with which it can be applied clinically. Our results suggest that elastic moduli measurements based on a subvolume from the trabecular bone region have significant predictive power for whole bone stiffness and trabecular bone stiffness (see Table 5). Moreover, elastic moduli measurements are independent of bone geometry and size and provide both uniaxial and shear moduli along all three axes. The small model size and accuracy make elastic moduli measurements based on HR-pQCT scans excellent indicators of trabecular bone quality in clinical applications.

This study has several limitations. First, HR-pQCT imaging of cadaver bone is not affected by patient motion artifacts commonly encountered under in vivo situations. The lack of surrounding soft tissue also may result in increased signal-to-noise ratio. However, a recent study on the reproducibility of HR-pQCT measurements indicated that error in microstructural and mechanical measurements owing to motion artifacts should be less than 4.5% in the distal tibia and distal radius.(49) Second, cortical and trabecular bone tissue properties were assumed to be constant and homogeneous for all the specimens. Therefore, the resulting mechanical measurements of µFE analysis reflect only the influence of bone microstructure and not intrinsic mineral quality. The mineralization of trabecular tissue is inhomogeneously distributed owing to bone resorption and formation. Thus the inclusion of inhomogeneity within the tissue modulus results might improve the prediction of apparent mechanical properties.(16,50) Furthermore, there is a potential for subject-specific cortical and trabecular tissue modulus to be derived from the equivalent HA densities of cortical and trabecular bone images. However, this requires the establishment of a calibrated relationship between tissue modulus and HA densities of HR-pQCT through experimentation.(51,52) Third, a global threshold technique provided by the HR-pQCT manufacturer was used to obtain the input image for voxel-based morphologic measurements and µFE analysis. A local adaptive threshold technique is expected to improve the voxel-based microstructural and mechanical measurements.(47) It will be of interest to test this technique on whole bone images of the distal tibia in future studies.

As a noninvasive high-resolution clinical imaging modality, HR-pQCT, has the potential to assess longitudinal changes in bone microstructure. All the microstructure parameters tested correlated significantly with their gold standards. Although some discrepancies were detected, the high correlations ensure the suitability of these HR-pQCT measurements for longitudinal assessment of bone quality. Nevertheless, osteoporosis can cause changes in the composition and distribution of bone tissue.(53) In several pathologic conditions, including osteomalacia and osteogenesis imperfecta, bone tissue density is undoubtedly altered. Such alterations could affect derived BV/TV^d^, which is based on the assumption that bone has a constant mineral density (1200 mg HA/cm^3^), as well as derived Tb.Th and Tb.Sp. Furthermore, the accuracy of HR-pQCT-based µFE analysis is also subject to mineral density changes of bone tissue because the input image relies on a fixed mineral density as a global threshold. It is known that several osteoporosis treatments are associated with altered mineralization density. To assess bone quality longitudinally in subjects under treatment for osteoporosis, it is critical to overcome the dependence of HR-pQCT measurements on mineral density changes.

In conclusion, the accuracy of microstructural and mechanical HR-pQCT measurements of the human distal tibia has been tested with reference to the current gold standard, high-resolution µCT. Differences between HR-pQCT and gold standard µCT measurements should be considered when intermodality comparisons are made. However, the high correlations we observed between patient and direct microstructural measurements and mechanical measurements of human distal tibiae suggest that HR-pQCT has the potential to become the new clinical standard for microstructural aspects of bone quality. Microstructural measurements and mechanical parameters of the distal tibia can be efficiently derived from 3D images of HR-pQCT scans and provide additional information regarding bone fragility.
